# α-Viniferin-Induced Apoptosis through Downregulation of SIRT1 in Non-Small Cell Lung Cancer Cells

**DOI:** 10.3390/ph16050727

**Published:** 2023-05-10

**Authors:** Cheng Huang, Zi-Jun Lin, Jui-Chieh Chen, Hao-Jun Zheng, Yu-Heng Lai, Hsiu-Chen Huang

**Affiliations:** 1 Department of Biotechnology and Laboratory Science in Medicine, National Yang Ming Chiao Tung University, Taipei 11221, Taiwan; 2 Center for Teacher Education, National Tsing Hua University, Hsinchu 30014, Taiwan; 3 Department of Applied Science, Nanda Campus, National Tsing Hua University, Hsinchu 30014, Taiwan; 4 Department of Biochemical Science and Technology, National Chiayi University, Chiayi City 60004, Taiwan; 5 Department of Chemistry, Chinese Culture University, Taipei 11114, Taiwan

**Keywords:** α-Viniferin, NCI-H460 cells, non-small cell lung cancer, apoptosis, nude mice

## Abstract

α-Viniferin, a natural stilbene compound found in plants and a polymer of resveratrol, had demonstrated potential anti-cancer and anti-inflammatory effects. However, the specific mechanisms underlying its anti-cancer activity were not yet fully understood and required further investigation. This study evaluated the effectiveness of α-viniferin and ε-viniferin using MTT assay. Results showed that α-viniferin was more effective than ε-viniferin in reducing the viability of NCI-H460 cells, a type of non-small cell lung cancer. Annexin V/7AAD assay results provided further evidence that the decrease in cell viability observed in response to α-viniferin treatment was due to the induction of apoptosis in NCI-H460 cells. The present findings indicated that treatment with α-viniferin could stimulate apoptosis in cells by cleaving caspase 3 and PARP. Moreover, the treatment reduced the expression of SIRT1, vimentin, and phosphorylated AKT, and also induced AIF nuclear translocation. Furthermore, this research provided additional evidence for the effectiveness of α-viniferin as an anti-tumor agent in nude mice with NCI-H460 cell xenografts. As demonstrated by the TUNEL assay results, α-viniferin promoted apoptosis in NCI-H460 cells in nude mice.

## 1. Introduction

Lung cancer is the primary and foremost cause of global cancer-related mortality. There are two primary types of lung cancer, small cell lung carcinoma (SCLC) and non-small cell lung carcinoma (NSCLC). NSCLC grows at a slower rate than SCLC and is generally considered to be less aggressive. NSCLC accounts for around 85% of all lung cancer cases and its 5-year survival is less than 18% [1]. Compared with other types of cancer, NSCLC is usually highly invasive and has relatively low sensitivity to chemotherapy drugs [2]. For these reasons, developing more effective and less toxic chemotherapeutic reagents for NSCLC is crucial for improving patient outcomes and reducing the side effects associated with chemotherapy. Additionally, the identification of new biomarkers capable of predicting the response of NSCLC patients to chemotherapy is crucial for enhancing their prognosis.

Sirtuins (SIRTs) are categorized as class III histone deacetylases (HDACs) and have seven family members (SIRT1-SIRT7) [3]. They serve as a critical regulator of both physiological and pathological periods including immune and inflammatory responses and cancer. SIRT1, an NAD^+^-dependent histone deacetylase, affects many biological systems through deacetylating a variety of proteins including histones and transcription factors. SIRT1 is a major player in the regulation of glucose and lipid metabolism in health and diseases. SIRT1 is also involved in a wide range of cellular processes directly linked to tumorigenesis, including genomic stability, DNA damage, and oxidative stress response. The role of SIRT1 in tumor progression remains controversial. Depending on the organ or even the species, SIRT1 has the ability to function as either a tumor suppressor or a tumor promoter. Many studies have demonstrated that SIRT1 was overexpressed in prostate, liver, lymphoma, colon, breast and gastric cancers [4,5]; and it is thought that inhibiting its activity could be a potential strategy for cancer treatment. Little is known about the roles of SIRT1 in NSCLC. Gharabaghi et al. reported that overexpression of SIRT1 was linked to poorer overall survival in patients with lung adenocarcinoma [6]. Thus, SIRT1 has been linked to the progression of tumors in NSCLC. Other research showed that depletion of SIRT1 could induce apoptosis and chemoresistance in NSCLC [4].

α-Viniferin is an oligostilbene of trimeric resveratrol, which is a class of polyphenolic compounds found in many plant varieties such as grapes [7]. α-Viniferin is one of the most abundant resveratrol oligomers found in grapes, and has been shown to have antioxidant, anti-inflammatory [8], and anti-cancer properties [9,10]. Previous research has highlighted the potential of α-viniferin as a therapeutic agent for various types of cancer, including colon cancer (HCT-116, HT-29, Caco-2) [11], promyelocytic leukemia (HL-60), oral squamous cell carcinoma (HSC-2) [12], prostate cancer (LNCaP, DU145 and PC-3) [9] and melanoma (SK-MEL-28) [13]. Additionally, our study revealed that α-viniferin can induce apoptosis in osteosarcoma cells (HOS and U2OS) and pneumonia cells (A549), as well as inhibit epithelial mesenchymal transition (EMT) in A549 lung cancer cells [10]. Its promising anti-cancer properties suggest great potential for cancer treatment. However, the mechanism by which α-viniferin exerts its anti-cancer effects is not fully understood. Small molecule SIRT1 inhibitors have been reported to show potential as anti-cancer drugs by inducing cancer cell death [14]. Resveratrol oligomers, including vitisin A, (−)-vitisin B, and (+)-hopeaphenol, have been found to have promising anti-cancer properties due to their ability to inhibit the activity of the human SIRT1 enzyme [15]. Therefore, these compounds have potential as a seed compound for the development of novel anti-cancer drugs. This study aimed to examine whether α-viniferin could affect the SIRT1 activity in NSCLC xenograft mode in vitro and in vivo.

## 2. Results

### 2.1. Impact of α-Viniferin and ε-Viniferin on Growth of NCI-H460 and A549 Cells

**Figure 1:**
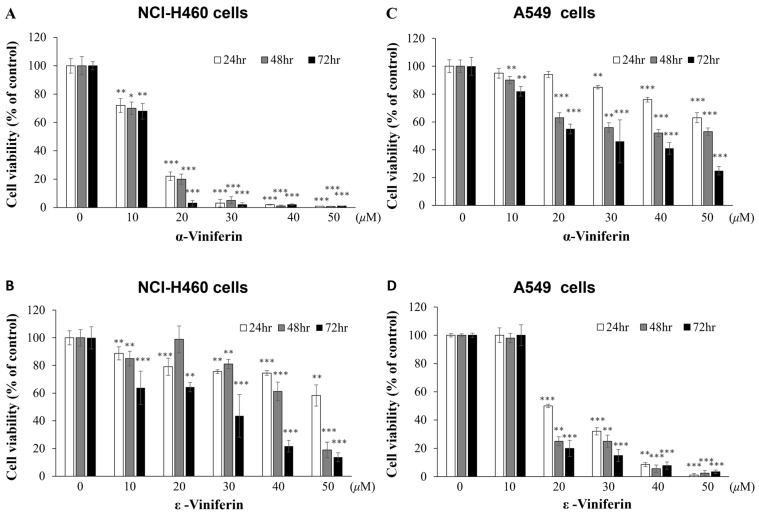
(**A**,**B**) The viability of NCI-H460 cells was assessed through an MTT assay after being exposed to different concentrations of α-viniferin (10, 20, 30, 40, and 50 μM) and ε-viniferin (10, 20, 30, 40, and 50 μM) for 24, 48, and 72 h. (**C**,**D**) Similarly, the viability of A549 cells was evaluated using the MTT assay after exposure to various concentrations of α-viniferin (10, 20, 30, 40, and 50 μM) and ε-viniferin (10, 20, 30, 40, and 50 μM) for 24, 48, and 72 h. Each value obtained from the assay was expressed as mean ± SD (*n* = 3). * indicates values significantly different from the control (*, *p* < 0.05; **, *p* < 0.01; ***, *p* < 0.001).

Both NCI-H460 and A549 cells are types of human large cell lung carcinoma and fall under the category of NSCLC subtypes. Cell viability was measured with MTT assay. The cell viability graphs (Figure 1) demonstrated that α-viniferin and ε-viniferin displayed unique anti-proliferative effects on both NCI-H460 and A549 cells. The data demonstrated that low doses of α-viniferin (10–20 μM) inhibited cell viability in a dose- and time-dependent manner in NCI-H460 cells, but not in A549 cells. However, in NCI-H460 cells, 20 μM of ε-viniferin did not exhibit potent anti-proliferative effects as α-viniferin did, while higher concentrations of ε-viniferin did demonstrate such effects. On the other hand, low doses of ε-viniferin (20 μM) inhibited A549 cell viability, while α-viniferin did not. Taken together, these results indicated greater effectiveness of α-viniferin than of ε-viniferin in suppressing cell proliferation in NCI-H460 cells, but not in A549 cells. Therefore, further studies should focus on the mechanism of α-viniferin on the effects of cell viability in NCI-H460 cells.

### 2.2. α-Viniferin-Induced Apoptosis in NCI-H460 Cells

**Figure 2:**
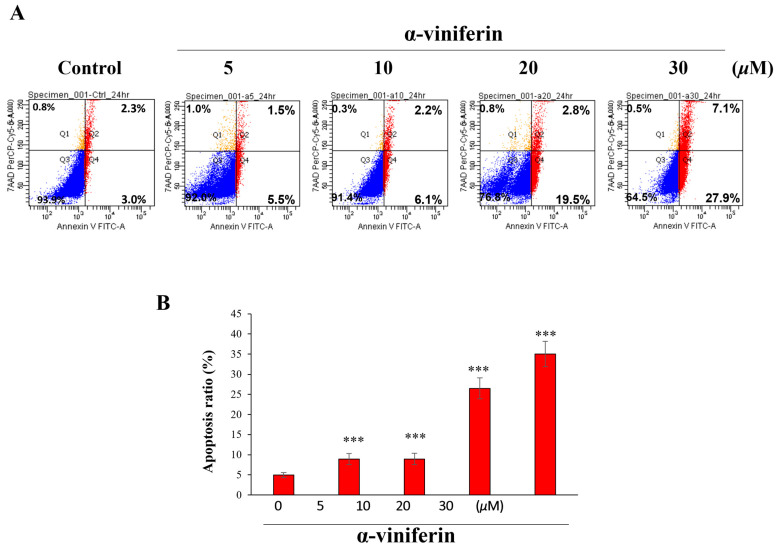
(**A**) α-Viniferin-induced apoptosis in NCI-H460 cells. After treatment with α-viniferin, the apoptotic rates of NCI-H460 cells were detected using flow cytometry. Images obtained from the flow cytometry were analyzed to determine the percentage of apoptotic cells after a 24-h incubation period with α-viniferin. “Red indicates apoptosis (annexin V+/7-AAD- or V+/7-AAD+), orange indicates necrosis (annexin V-/7-AAD+), and blue indicates viability (annexin V-/7-AAD-)”. (**B**) The total percentage of apoptotic cells was calculated as the combined percentage of early and late apoptotic cells. * indicates values significantly different from the control (***, *p* < 0.001).

To investigate the molecular mechanism by which α-viniferin affects NCI-H460 cells, the presence of apoptotic and necrotic cells was analyzed using double staining with annexin V and 7-aminoactinomycin D (7-AAD). Annexin V, which binds to phosphatidylserine (PS), is used as a marker of early-stage apoptosis. The 7-ADD is a fluorescent DNA dye that is excluded from viable cells with intact membranes but can penetrate the plasma membrane of apoptotic and necrotic cells. Double staining with both annexin V and 7-AAD can differentiate between cells in different stages of apoptosis and necrosis. Specifically, cells that are annexin V+/7-AAD- are in early apoptosis, cells that are annexin V+/7-AAD+ are in late apoptosis, and cells that are annexin V-/7-AAD+ are in necrosis. Cells that are annexin V-/7-AAD- are considered viable. Treatment of NCI-H460 cells with various concentrations of α-viniferin (0 μM, 5 μM, 10 μM, 20 μM, and 30 μM) for 24 h resulted in the presence of 5.3%, 7.0%, 8.3%, 22.3%, and 35.0% of apoptotic cells (early + late apoptotic cells), respectively (Figure 2). These findings implied dose-dependent induction of apoptosis by α-viniferin in NCI-H460 cells. The mechanism behind α-viniferin-induced apoptosis in NCI-H460 cells merits further investigation.

### 2.3. α-Viniferin-Induced Caspase-3 Activation, and PARP Cleavage, and Reduced SIRT1, Vimentin, and AKT Phosphorylation in NCI-H460 Cells

**Figure 3:**
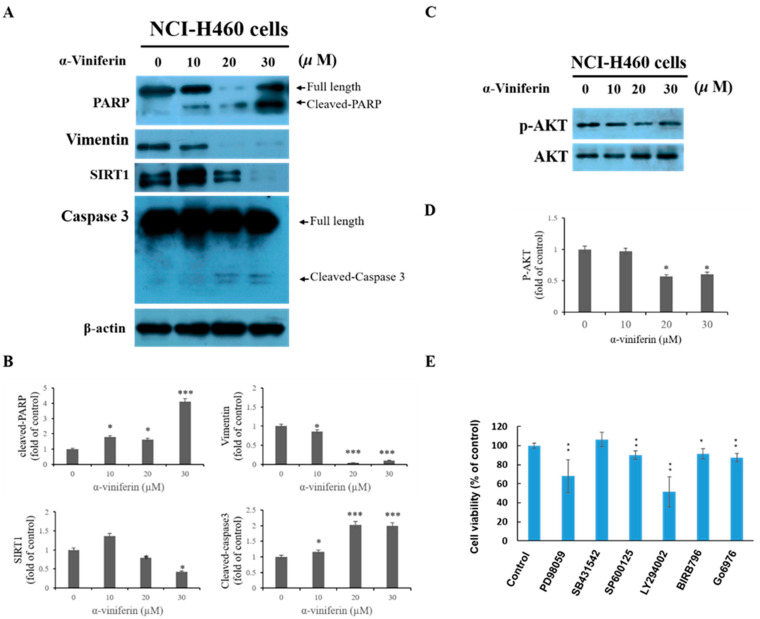
Western blot analysis of α-viniferin effect on apoptosis-related expression of proteins, including cleaved caspase-3, cleaved PARP, SIRT1, vimentin (**A**,**B**), and AKT phosphorylation (**C**,**D**). The protein levels were normalized to specific reference proteins, such as β-actin for cleaved caspase-3, cleaved PARP, SIRT1, and vimentin, and AKT for AKT phosphorylation. Data are expressed as mean ± SD. (**E**) NCI-H460 cell viability was evaluated using MTT assay after treatment with various concentrations of signal transduction inhibitors (20 μM PD98059, 10 μM SB431542, 20 μM SP600125, 20 μΜ LY294002, 10 μM BIRB796, or 10 μM GO6976) for 24 h. * indicates values significantly different from the control (*, *p* < 0.05; **, *p* < 0.01; ***, *p* < 0.001).

Caspase 3 is a member of the cysteine protease family and is known to play a critical role in apoptosis. Poly (ADP-ribose) polymerase (PARP) is an enzyme located in the nucleus that participates in the processes of DNA repair and apoptosis. During apoptosis, PARP is cleaved by caspases 3, resulting in the formation of a truncated PARP fragment. A previous study has suggested that knockdown of SIRT1 in NCI-H460 cells leads to a decrease in cell viability and induces apoptosis [16]. To further investigate the mechanisms involved in α-viniferin-induced apoptosis in NCI-H460 cells, western blot analysis was performed to determine the protein levels of activated caspase-3, cleaved PARP, SIRT1, vimentin, and AKT phosphorylation. The results demonstrated that α-viniferin increased the protein expression of activated caspase-3 and cleaved PARP (Figure 3A,B). Additionally, α-viniferin decreased the protein expression levels of SIRT1, vimentin, and AKT phosphorylation in NCI-H460 cells (Figure 3A–D). The intracellular signaling pathways of AKT, mitogen-activated protein kinase (MAPK), and TGF-β have been extensively studied for their crucial roles in regulating various processes associated with carcinogenesis, including cell proliferation, cell cycle progression, and apoptosis [17,18]. Furthermore, the cell viability of NCI H460 cells was analyzed in the presence of various signal transduction pathway inhibitors. NCI H460 Cells were exposed to six different inhibitors for 24 h: LY294002 (an AKT inhibitor) at a concentration of 20 μM, PD98059 (an ERK1/2 inhibitor) at 20 μM, SP600125 (a JNK1/2 inhibitor) at 20 μM, BIRB796 (a p38 inhibitor) at 10 μM, SB431542 (a TGF-β RI Kinase inhibitor) at 10 μM, and GO6976 (a PKC inhibitor) at 10 μM. Following treatment, cell viability was measured using the MTT assay. The current findings revealed that blocking the signaling pathway via AKT with LY294002 and ERK with PD98059 decreased cell viability in NCI-H460 cells (Figure 3E).

### 2.4. α-Viniferin-Induced AIF Translocation and Deoxyribonucleic Acid (DNA) Fragmentation

**Figure 4:**
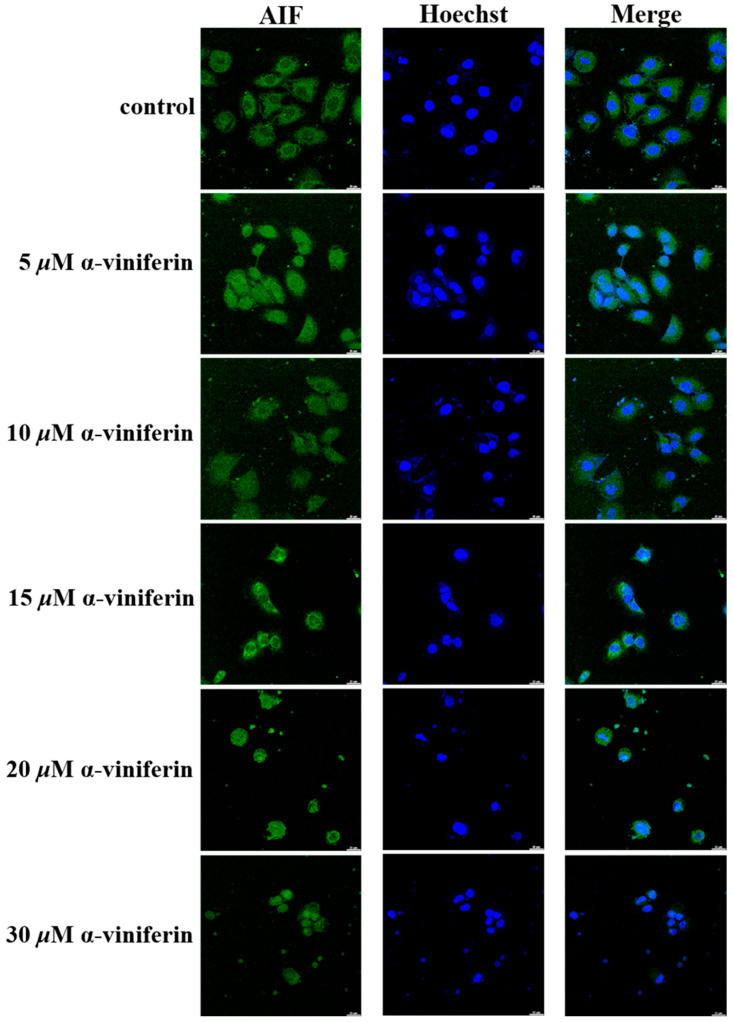
Translocation of AIF to the nucleus after treatment with α-viniferin investigated using immunofluorescence assay. Cells were exposed to different concentrations of α-viniferin (5, 10, 15, 20, and 30 μM) for a duration of 24 h. After incubation, the cells were stained with AIF antibody (green) and Hoechst 33258 (blue). The resulting images were observed under confocal laser scanning microscopy, with a bar representing a length of 20 μm.

Apoptosis can be induced through both caspase-dependent and caspase-independent mechanisms. Apoptosis-inducing factor (AIF), a flavoprotein located in the mitochondria, played an important role in caspase-independent cell death. AIF translocated to the nucleus during the process of apoptosis and triggered nuclear chromatin condensation and DNA fragmentation [19]. α-Viniferin induced apoptosis by causing mitochondrial dysfunction and subsequent release of AIF, which then translocated to the nucleus and caused DNA fragmentation in NCI-H460 cells (Figure 4). The results of this study suggest that α-viniferin treatment can induce apoptosis in cells via both caspase-dependent mechanisms involving caspase 3 activation, and caspase-independent pathways through the release of AIF from the mitochondria. 

### 2.5. Effects of α-Viniferin on Tumor Growth In Vivo

**Figure 5:**
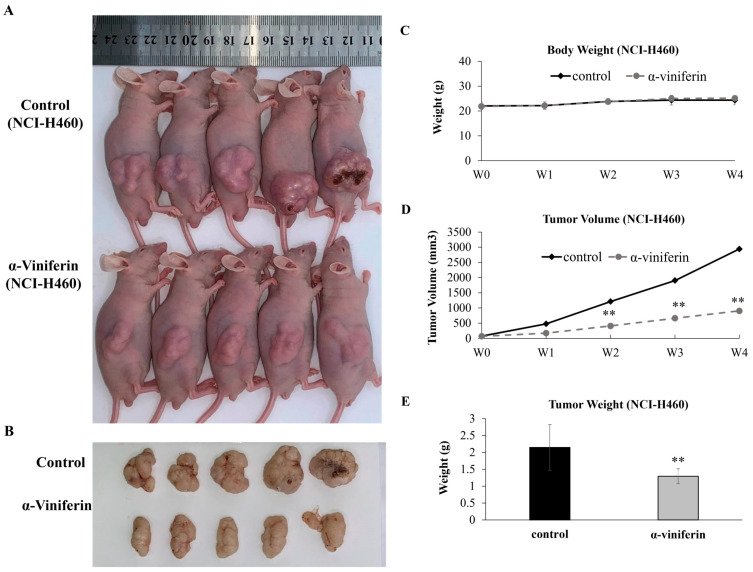

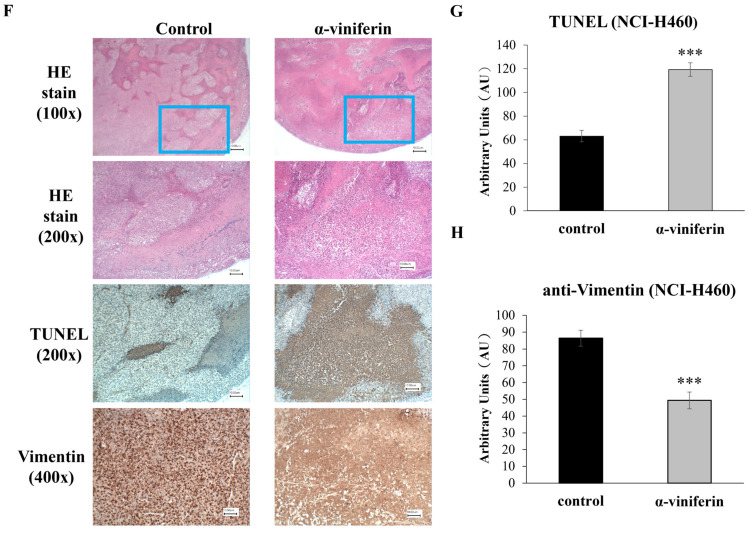

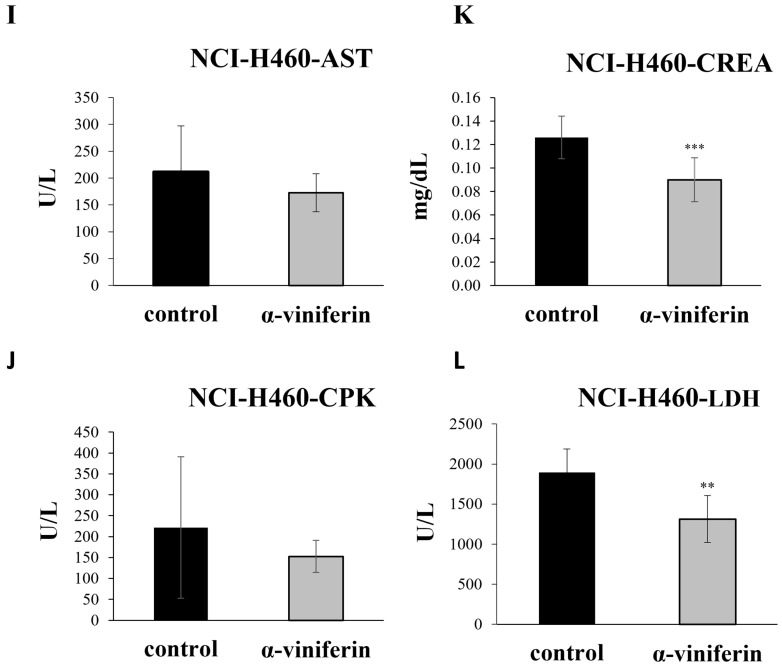
Effect of α-viniferin on tumor growth in NCI-H406 cells. (**A**) Representative images of NCI-H406 cell xenograft-bearing nude mice (upper panel) and (**B**) tumors (lower panel), and (**C**) body weights, (**D**) tumor volumes, and (**E**) tumor weights determined in mice xenografted with NCI-H460 cells. (**F**–**H**) Tumor morphology, vimentin, TUNEL signal in NCI-H406 cell xenograft-bearing nude mice were examined using H&E and IHC staining (original magnification ×200–400) with quantitative analysis of vimentin and TUNEL signal performed using Image J. The bar in the images represents a length of 10 μm. (**I**–**L**) Finally, AST, LDH, CPK, and creatinine levels in serum were determined. All values were expressed as mean ± SD. * indicates values significantly different from the control (**, *p* < 0.01; ***, *p* < 0.001).

Furthermore, to investigate whether α-viniferin inhibited tumor growth in vivo, this study also generated a nude mice xenograft tumor model using NCI-H460 cells. Compared with the control treatment, α-viniferin treatment resulted in more significant reduction in tumor weight and tumor volume in NCI-H460-cell xenograft-bearing nude mice (Figure 5A–E). Terminal deoxynucleotidyl transferase-mediated dUTP nick end-labeling (TUNEL) staining was conducted to detect cells undergoing apoptosis in the tumor tissue and showed increased numbers of apoptotic cells in tumor tissue in the α-viniferin-treated groups, but not in the control group (Figure 5F,G). The levels of vimentin expression were reduced in the groups treated with α-viniferin as compared with the control group (Figure 5F,H). To assess any potential side effects of α-viniferin, aspartate aminotransferase (AST), creatine phosphokinase (CPK), lactic dehydrogenase (LDH), and creatinine values were measured. The results depicted in Figure 5I–L demonstrated no negative impact of α-viniferin on liver or kidney function in the mice. Overall, these findings provide further evidence for the in vivo anti-tumor effects of α-viniferin.

## 3. Discussion

NSCLC is one of the most common and deadly cancers worldwide. NSCLC cells are highly invasive and often less sensitive to chemotherapy, making it challenging to control the cancer. Therefore, the search for new chemotherapeutic agents that are both highly effective and less toxic in the treatment of NSCLC is of utmost importance. SIRT1 is a protein that has been demonstrated to participate in a range of cellular processes, such as regulating the cell cycle, repairing DNA damage, and metabolism. It has also been implicated in cancer, as it can affect tumor growth and survival. Previous findings indicated that SIRT1 played a crucial role in controlling the viability and apoptosis of NCI-H460 cells [16]. Additionally, preclinical investigations have also demonstrated that SIRT 1 inhibitors could reduce the proliferation of cancer cells, induce apoptosis, and enhance the sensitivity of cancer cells to chemotherapy and radiation therapy [20]. 

Previous studies have shown that SIRT1 could inhibit apoptosis by deacetylating the p53 protein [21,22]. p53 was an important tumor suppressor protein that played a role when cellular DNA was damaged. The deacetylation of p53 by SIRT1 reduced in a reduction in its effectiveness, enabling cancer cells to resist apoptosis. Therefore, inhibition of SIRT1 or activation of p53 may be a potential strategy for treating cancer. To our current knowledge, no previous research has demonstrated that α-viniferin can regulate the activity of p53. Therefore, further research is needed to determine whether α-viniferin can inhibit SIRT1 and further inactivate or regulate p53 activity in the NCI-H460 cell line.

Silencing SIRT1 not only induced apoptosis in cancer cells but also caused a significant reduction in their migration and invasion abilities [23,24,25]. SIRT1 can deacetylate p53 and vimentin proteins to regulate cell apoptosis or cell invasion and migration, respectively. Vimentin, a type III intermediate filament protein, is typically found in mesenchymal cells and is often overexpressed in cancer cells. Its overexpression has been associated with the promotion of tumor cell migration and invasion. Liao et al. demonstrated that silencing SIRT1 decreased vimentin and N-cadherin expression levels, which, in turn, reduced the number of migrating and invasive A549 cells [26]. Recent research had also indicated that cells lacking vimentin were more likely to undergo apoptosis [27]. However, further research was needed to confirm this mechanism. Our previous research had confirmed that α-viniferin could inhibit TGF-induced vimentin expression and thus inhibited epithelial-mesenchymal transition (EMT) in NSCLC cells [28]. The present results further supported that α-viniferin inhibited both SIRT1 and vimentin expressions in NCI-H460 cells. However, the detailed mechanism by which α-viniferin regulated the relationship between SIRT1 and vimentin still needed further investigation. 

EGFR overexpression or mutations in the intracellular EGFR are frequently observed in NSCLC patients, with a prevalence of 43–89% [29,30]. These mutations can cause aggressive tumor growth, metastasis, and resistance to cancer drugs. In Asia, activating mutations in exons 18–21 of the EGFR tyrosine kinase domain occur in around 50% of NSCLC cases, resulting in the constant activation of signaling pathways that promote cell proliferation and inhibit apoptosis, independent of extracellular ligands [31]. Therefore, targeting EGFR has become a crucial strategy for NSCLC treatment. Various NSCLC cell lines have been identified, including EGFR wild-type cell lines (H522, H358, H460, A549, and H1299) and EGFR-mutated cell lines (H1975, H1650, and HCC827). While both H460 and A549 cell lines used in this study are wild-type for EGFR, there has been no research conducted to evaluate the impact of α-viniferin and ε-viniferin on NSCLC cells that have mutated EGFR. Therefore, further exploration of the effects of α-viniferin and ε-viniferin on EGFR-mutated NSCLC cells is necessary. One effective approach is using epidermal growth factor receptor tyrosine kinase inhibitors (EGFR-TKIs) that hinder the proliferation of lung cancer cells. A recent study has shown that SIRT1 can selectively eliminate EGFR TKI-resistant cancer stem cells by regulating mitochondrial oxidative phosphorylation in lung adenocarcinoma [32]. Therefore, further research is needed to determine if α-viniferin can also inhibit EGFR-mutated NSCLC cells by suppressing SIRT1. Finally, understanding the effects of α-viniferin and ε-viniferin on both EGFR wild-type and EGFR-mutated NSCLC cells could have significant clinical implications.

In summary, the present study is the first to demonstrate that α-viniferin can regulate SIRT1-induced apoptosis. α-Viniferin was found to inhibit SIRT1, vimentin, and AKT phosphorylation, while increasing cleavage of caspase 3 and PARP, leading to activation of the caspase-dependent pathway and the AIF-mediated caspase-independent pathway. Furthermore, administration of α-viniferin (5 mg/kg, i.p., five days/week) in vivo resulted in the suppression of tumor growth, and decreased TUNEL positive cells and vimentin expression in nude mice bearing NCI-H460 cells. Further preclinical and clinical studies are needed to evaluate the potential of α-viniferin as novel therapeutic agents for NSCLC. 

## 4. Materials and Methods

### 4.1. Chemicals 

α-Viniferin (CAS Number 62218-13-7, Purity: ≧98%) was purchased from SunHank Technology (Tainan City, Taiwan). [3-(4, 5-dimethylthiazol-2-yl) -2,5-diphenyl tetrazolium bromide] (MTT) (CAS Number: 298-93-1), LY294002 (CAS Number 154447-36-6, Purity: ≧99%), PD 98,059 (CAS Number 167869-21-8, Purity: ≧99%), SB431542 (CAS Number 301836-41-9, Purity: ≧99%), SP600125 (CAS Number 129-56-6, Purity: ≧99%), BIRB796 (CAS Number 285983-48-4, Purity: ≧99%), GO6976 (CAS Number 154675-18-0, Purity: ≧99%), and ε-viniferin (CAS Number 62218-08-8, Purity: ≧98%) were purchased from Sigma-Aldrich (St. Louis, MO, USA). Anti-AKT antibody (GTX121937), anti-AKT (phospho Ser473) antibody (GTX128414), Anti-β-actin antibody (GTX109639), anti-vimentin, anti-SIRT1 (GTX17532), anti-AIF (GTX60476), anti-PARP (GTX100573) and anti-caspase 3 (GTX110543) antibody were purchased from GeneTex (Irvine, CA, USA). 

### 4.2. Cell Culture

The Bioresource Collection and Research Center (BCRC) in Hsinchu, Taiwan provided the NCI-H460 and A549 cell lines, which were cultured in DMEM supplemented with 10% FBS, 100 U/mL penicillin A, and 100 U/mL streptomycin. These cells were kept at a temperature of 37 °C with 5% CO_2_ and a humidified air environment.

### 4.3. Cell Viability Assay 

To investigate the impact of α-viniferin (0–50 μM), ε-viniferin (0–50 μM), and signal transduction inhibitors (20 μM PD98059, 10 μM SB431542, 20 μM SP600125, 20 μΜ LY294002, 10 μM BIRB796, or 10 μM GO6976) on the viability of NCI-H460 or A549 cells were investigated using MTT assay. NCI-H460 or A549 cells were treated with various concentrations of α-viniferin, ε-viniferin, and signal transduction inhibitors and were incubated for 24, 48, or 72 h. Following incubation, 5 mg/mL MTT solution in phosphate-buffered saline (PBS) was added to each well, and the plate was further incubated for 4 h at 37 °C. Following this, the medium was aspirated, and DMSO was utilized to dissolve the MTT-formazan crystals. Finally, the solution was analyzed using a microplate reader at a wavelength of 540 nm.

### 4.4. Flow Cytometry Analysis

The annexin V and 7-ADD double staining was utilized to detect apoptotic and necrotic cells. NCI-H460 cells were treated with α-viniferin at various concentrations (0, 5, 10, 20, and 30 μM) for 24 h. Following treatment, the cells were collected by trypsinization and suspended in a warm medium. The cells in a suspension were centrifuged at 2000× *g* for 5 min, and the resulting supernatant was removed, leaving the cell pellets. The pellets were subsequently resuspended in 200 µL of PBS and were fixed by adding 800 µL of 100% ethanol at −20 °C. Next, the cell pellets that had been fixed were resuspended in 500 µL of 1 × annexin V-FITC Binding Buffer, to which 5 µL each of annexin V-FITC and 7-AAD staining reagents were added. The samples were then vortexed gently and incubated for 15 min at 4 °C in the dark. Finally, the samples were analyzed using a BD FACScan™ flow cytometer.

### 4.5. Western Blot Analysis

The western blot analysis was conducted to check the expression levels of cleaved caspase-3, cleaved PARP, SIRT1, vimentin, and AKT phosphorylation in NCI-H460 cells after treatment with α-viniferin at various concentrations (0, 10, 15, 20, and 30 μM) for 24 h. After treatment, the cells in a 10-cm plate were lysed in an ice-cold lysis buffer, containing protease and phosphate inhibitors. The Bio-Rad protein assay kit (Bio-Rad Laboratories, Hercules, CA, USA) was utilized to measure the protein concentration in each lysate. Equal amounts of protein (50 μg) were loaded onto each lane, separated using sodium dodecyl sulfate-polyacrylamide gel electrophoresis (SDS-PAGE), and subsequently transferred onto polyvinylidene fluoride (PVDF) membranes. First, the membranes were blocked using 1% BSA in PBS at room temperature for 1 h. Then, the membranes incubated with primary antibodies against full length caspase-3, activated caspase-3, full length PARP, cleaved PARP, SIRT1, vimentin, and AKT phosphorylation, and followed by secondary antibodies conjugated with horseradish peroxidase were added to the samples. Finally, the immunoreactive bands were visualized using an enhanced chemiluminescence (ECL) detection system. 

### 4.6. Immunofluorescence Assay 

The immunofluorescence assay was conducted following the method described in a previous study to detect the localization of AIF in NCI-H460 cells after treatment with α-viniferin at various concentrations (0, 5, 10, 15, 20, and 30 μM) for 24 h [28]. After treatment, the cells in a 6-well plate were washed with PBS, and fixed immediately with 4% formaldehyde at room temperature for 15 min. Then, NCI-H460 cells were treated with 0.1% Triton X-100 in PBS at room temperature for 15 min to achieve permeabilization. After that, NCI-H460 cells were incubated with anti-AIF primary antibodies at 4 °C for 24 h, followed by incubation with secondary antibodies conjugated with fluorescein isothiocyanate at 37 °C for 1 h. To stain the cell nuclei, NCI-H460 cells were treated with a solution containing 4 mg/mL Hoechst 33,258 for 30 min. After washing the cells with PBS, they were mounted onto a glass slide for visualization. Confocal laser scanning microscopy was employed to capture the images. The mean fluorescence intensity, which represented the protein level, was analyzed using the Image J software.

### 4.7. In Vivo Xenograft Tumor Growth Experiments

The male BALB/c nude mice utilized in this investigation were obtained from the National Laboratory Animal Breeding and Research Center situated in Taiwan. All animal experimentation procedures were conducted in accordance with National Tsinghua University’s Institutional Animal Care and Use Committee Guidelines (Protocol No. 107049). For this study, the hind leg of five-week-old nude mice was subcutaneously implanted with 1 × 10^6^ NCI-H460 cells. Treatment was initiated only after the tumors had grown to a volume of 100–200 mm^3^. Subsequently, the nude mice with NCI-H460-cell xenografts were randomly allocated into two groups: experimental and control, each comprising five mice. The experimental group was administered 5 mg/kg α-viniferin through intraperitoneal injection five times every week, while the control group was administered PBS. After four weeks, the mice were euthanized. Tumor weight, vimentin expression, histopathological changes and apoptosis in the tumor section were evaluated using immunohistochemistry (IHC) staining, hematoxylin and eosin staining, and TUNEL assay. The quantification of AST, CPK, LDH, and creatinine levels was carried out following previously described procedures [10]. Briefly, AST, LDH, CPK, and creatinine levels were quantified using commercial kits (Wako Pure Chemical Industries, Osaka, Japan).

### 4.8. Statistical Analysis

Mean ± SD values were used to present the results, with each value being the average of at least three independent experiments per group. Statistical analysis was conducted using the Student’s *t*-test, and significant differences from the control treatment were indicated by asterisks, with * denoting *p* < 0.05, ** denoting *p* < 0.01, and *** denoting *p* < 0.001.

## Data Availability

Not applicable.
